# Compensatory Alignment Characteristics of Coronal Spinopelvic and Lower Leg Alignments in Patients With Crowe Type IV Developmental Dysplasia of the Hip

**DOI:** 10.7759/cureus.104715

**Published:** 2026-03-05

**Authors:** Satomi Nagamine, Takaomi Kobayashi, Tatsuya Sakai, Toshihiro Nonaka, Yosuke Matsumura, Tadatsugu Morimoto

**Affiliations:** 1 Department of Orthopaedic Surgery, Saga University, Saga, JPN

**Keywords:** cobb angle, crowe type iv, developmental dysplasia of the hip, leg length discrepancy, pelvic inclination

## Abstract

Introduction

We aimed to investigate compensatory alignment characteristics of coronal spinopelvic and lower limb alignments in patients with unilateral Crowe type IV developmental dysplasia of the hip (DDH).

Methodology

We conducted a cross-sectional study of 87 patients with unilateral Crowe type IV DDH at our hospital between September 2005 and August 2015. Using standing anteroposterior radiographs of the thoracic and lumbar spine, pelvis, and lower limbs, the inter-ankle height difference, mechanical axis percentage (%MA), leg length discrepancy, pelvic inclination, and Cobb angle were assessed. Spearman’s rank correlation coefficient (ρ) was used to evaluate the associations between the inter-ankle height difference and other coronal spinopelvic and lower limb alignment parameters. For the relationships between the inter-ankle height difference and each alignment, multivariable linear regression analysis was performed to calculate adjusted β coefficients, controlling for age, sex, and body mass index. The inter-ankle height difference was treated as the dependent variable, and the other alignments were treated as independent variables.

Results

The mean age was 65.1 ± 8.6 years, and 77 patients (88.5%) were female. Spearman’s rank correlation analysis demonstrated a significant positive weak correlation between the inter-ankle height difference and pelvic inclination (ρ = 0.335, p = 0.002). The inter-ankle height difference showed marginal significant weak association with %MA (ρ = 0.206, p = 0.055) and the Cobb angle (ρ = 0.192, p = 0.075). In contrast, no significant correlation was observed between the inter-ankle height difference and leg length discrepancy (ρ= 0.039, p = 0.721). In multivariate linear regression analysis, the inter-ankle height difference was significantly associated with pelvic inclination (adjusted β = 1.100, 95% CI 0.532-1.669) and Cobb angle (adjusted β = 0.330, 95% CI 0.039-0.620). No significant associations were observed with %MA or leg length discrepancy.

Conclusion

This cross-sectional study demonstrated that ankle-level compensation was significantly associated with pelvic inclination and showed a borderline association with spinal alignment in patients with unilateral Crowe type IV DDH, whereas knee alignment showed no robust association. These findings suggest that compensatory alignment changes for long-standing leg length discrepancy tend to be observed at the pelvic level, with secondary involvement of the spine rather than the knee.

## Introduction

Unilateral Crowe type IV developmental dysplasia of the hip (DDH) is characterized by complete dislocation of the femoral head and proximal migration exceeding 100% and severe and long-standing leg length discrepancy [1]. In adult patients, this asymmetry often persists for decades before surgical intervention and requires various compensatory adaptations across the musculoskeletal system to maintain standing balance and gait [2]. These compensatory responses may involve multiple segments along the kinetic chain, including the ankle, knee, pelvis, and spine [3].

Previous studies have reported associations between leg length discrepancy and secondary changes in pelvic alignment, spinal curvature, and lower limb loading [3-6]. Pelvic obliquity and scoliosis are common findings in patients with severe unilateral hip dislocation [1-3]. However, the relative contribution of different joints to compensation, and how compensatory demands are distributed across distal and proximal segments, remain incompletely understood. Most prior investigations have focused on proximal alignment changes, such as the pelvis or spine, without simultaneously evaluating distal compensatory mechanisms [1-3,7].

Ankle-level compensation, potentially including equinus positioning, represents an important potential mechanism for adjusting functional leg length but has been insufficiently studied in patients with unilateral Crowe type IV DDH. From a biomechanical standpoint, compensation at the ankle directly alters effective leg length and may influence alignment at more proximal joints [3]. The aim of the present study was to investigate the association between ankle-level compensation and coronal spinopelvic and lower limb alignment in patients with unilateral Crowe type IV DDH.

## Materials and methods

Study design

**Figure 1:**
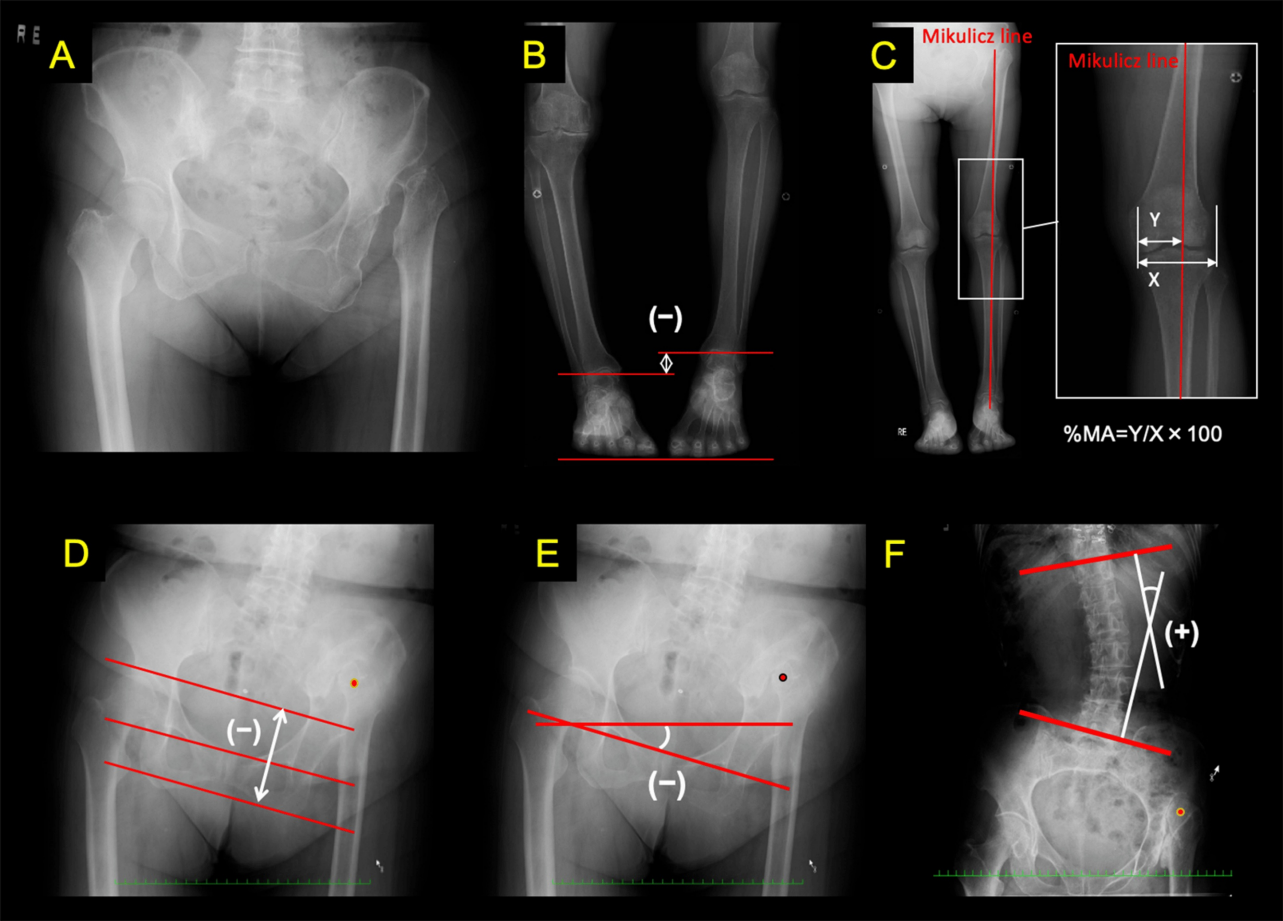
Unilateral Crowe type IV developmental dysplasia of the hip (DDH) (A), the inter-ankle height difference (B), mechanical axis percentage (%MA) (C), leg length discrepancy (D), pelvic inclination (E), and Cobb angle (F)

This cross-sectional study included patients who underwent total hip arthroplasty for unilateral Crowe type IV DDH at Saga University Hospital between September 2005 and August 2015. Eligible patients had no history of knee or ankle surgery and were classified as Crowe type IV according to the Crowe classification, which categorizes developmental dysplastic hips based on the degree of femoral head subluxation. Crowe type IV is defined as greater than 100% subluxation (Figure 1A) [1].

Among 91 patients who underwent total hip arthroplasty for unilateral Crowe type IV DDH during the study period, four were excluded because of a history of knee or ankle surgery. No patients had a history of spinal surgery. Consequently, a total of 87 patients were included in the final analysis.

The study protocol adhered to the ethical principles of the 1975 Declaration of Helsinki and was approved by the institutional review board of our hospital (approval number: 2019-12-R-06). Informed consent was waived because of the retrospective nature of the study. Instead, an opt-out approach was adopted, whereby information regarding the study and data usage was publicly disclosed, and participants were given the opportunity to decline the use of their anonymized data. All participant information was anonymized to ensure confidentiality.

Outcome of interest

Demographic and anthropometric characteristics, including age, sex, and body mass index, were retrospectively collected from preoperative medical records.

Coronal spinopelvic and lower limb alignment parameters included the inter-ankle height difference (Figure 1B) [8], mechanical axis percentage (%MA) (Figure 1C) [4], leg length discrepancy (Figure 1D) [5], pelvic inclination (Figure 1E) [6], and Cobb angle (Figure 1F) [6]. These parameters were evaluated using preoperative standing anteroposterior radiographs of the thoracic and lumbar spine, pelvis, and lower limbs. During radiographic acquisition, patients were instructed to stand barefoot without shoe lifts, with both knees fully extended, in a relaxed and natural upright posture. All radiographs were obtained according to the standard protocol at our institution as previously reported [6].

The inter-ankle height difference was measured on a standing anteroposterior radiograph of the lower limbs as the difference between two lines drawn parallel to the floor through the midpoint of the tibiotalar joint, defined as the center of the ankle joint space (Figure 1B) [8]. In this study, a negative value was assigned when the ankle joint on the side of the affected hip was lower than that on the contralateral side. The %MA was calculated as the ratio of the distance from the medial edge of the tibial plateau to the intersection point of the Mikulicz line (Y) divided by the total width of the tibial plateau (X), multiplied by 100 (Figure 1C) [4]. The mechanical axis of the lower limb (Mikulicz line) was defined as the line connecting the center of the femoral head and the center of the ankle joint [4]. Leg length discrepancy was measured on an anteroposterior pelvic radiograph as the vertical distance between the tips of the bilateral lesser trochanters, using a reference line drawn parallel to the inter-teardrop line (Figure 1D) [5]. In this study, a negative value indicated that the lower limb on the side of the affected hip was shorter. Pelvic inclination was measured on an anteroposterior pelvic radiograph, with downward inclination of the pelvis on the side of the affected hip defined as a positive value (Figure 1E) [6]. The Cobb angle was measured on an anteroposterior spinal radiograph, with convexity toward the side of the affected hip defined as a positive value (Figure 1F) [6].

Statistical analysis

All radiographs were digitally calibrated prior to measurement using the built-in measurement software to account for magnification effects. The coronal spinopelvic and lower limb alignment parameters were independently measured on separate days by two experienced orthopedic surgeons (SN and TN), who were blinded to clinical information. Inter-observer reliability (ICC) was assessed using ICC. The ICC values were 0.999 for inter-ankle height difference, 0.993 for %MA, 0.824 for leg length discrepancy, 0.838 for pelvic inclination, and 0.995 for Cobb angle, indicating good to excellent reliability [9].

The normality of the distribution of quantitative variables was assessed using the Shapiro-Wilk test, which indicated that all coronal spinopelvic and lower limb alignment parameters were non-normally distributed. Accordingly, Spearman’s rank correlation coefficient (ρ) was used to evaluate the associations between the inter-ankle height difference and other coronal spinopelvic and lower limb alignment parameters. Correlation coefficients of 0.00-0.10, 0.10-0.39, 0.40-0.69, 0.70-0.89, and 0.90-1.00 were defined as negligible, weak, moderate, strong, and very strong correlation, respectively [7,10].

In addition, simple linear regression analysis was performed to estimate crude regression coefficients (β) for the relationships between the inter-ankle height difference and each alignment. Multivariable linear regression analysis was subsequently conducted to calculate adjusted β coefficients, controlling for age (years, continuous), sex (0 = male, 1 = female), and body mass index (kg/m², continuous). The inter-ankle height difference (mm, continuous) was treated as the dependent variable, and the other coronal spinopelvic and lower limb alignments were treated as independent variables.

No missing data were identified in the study variables; therefore, complete-case analysis was performed. A two-sided p-value < 0.05 was considered statistically significant. R statistical software version 4.5.0 (R Foundation for Statistical Computing, Vienna, Austria) was used for the ICC. All other statistical analyses were performed using JMP® Student Edition version 18 (SAS Institute Inc., Cary, NC).

## Results

Patient characteristics

A total of 87 patients were included in the analysis. The mean age was 65.1 ± 8.6 years, and 77 patients (88.5%) were female. The mean body mass index was 23.7 ± 3.7 kg/m², and the affected side was the right hip in 38 patients (43.7%). The mean inter-ankle height difference was -23.9 ± 21.8 mm, indicating that the ankle joint on the side of the affected hip was, on average, positioned lower than that on the contralateral side. The mean %MA was 67.6 ± 29.2. The mean leg length discrepancy was -61.4 ± 16.0 mm. The mean pelvic inclination was 8.4 ± 6.5°, and the mean Cobb angle was 16.1 ± 13.2° (Table 1).

**Table 1:** Patient demographics %MA, mechanical axis percentage

Characteristic	Value (n = 87)
Age, years	65.1 ± 8.6
Female, n (%)	77 (88.5)
Body mass index, kg/m²	23.7 ± 3.7
Right affected side, n (%)	38 (43.7)
Inter-ankle height difference, mm	-23.9 ± 21.8
%MA	67.6 ± 29.2
Leg length discrepancy, mm	-61.4 ± 16.0
Pelvic inclination, degrees	8.4 ± 6.5
Cobb angle, degrees	16.1 ± 13.2

Correlation analysis

Spearman’s rank correlation analysis demonstrated a significant positive weak correlation between the inter-ankle height difference and pelvic inclination (ρ = 0.335, p = 0.002). The inter-ankle height difference was not significantly correlated with %MA (ρ = 0.206, p = 0.055) or the Cobb angle (ρ = 0.192, p = 0.075). In contrast, no significant correlation was observed between the inter-ankle height difference and leg length discrepancy (ρ = 0.039, p = 0.721) (Table 2).

**Table 2:** Spearman’s correlation between the inter-ankle height difference and the other coronal spinopelvic and lower limb alignments in patients with Crowe type IV DDH (n = 87) DDH, developmental dysplasia of the hip; %MA, mechanical axis percentage

Parameter	Spearman’s correlation coefficient	p-value
%MA	0.206	0.055
Leg length discrepancy	0.039	0.721
Pelvic inclination	0.335	0.002
Cobb angle	0.192	0.075

A significant positive moderate correlation was observed between the pelvic inclination and Cobb angle (ρ = 0.416, p < 0.001).

Regression analysis

In univariate linear regression analysis, the inter-ankle height difference was significantly associated with pelvic inclination (crude β = 1.473, 95% CI 0.824-2.123) and Cobb angle (crude β = 0.400, 95% CI 0.053-0.748). No significant associations were observed with %MA or leg length discrepancy.

After adjustment for age, sex, and body mass index, pelvic inclination remained significantly associated with the inter-ankle height difference (adjusted β = 1.100, 95% CI 0.532-1.669). The association between the inter-ankle height difference and Cobb angle also remained statistically significant after adjustment (adjusted β = 0.330, 95% CI 0.039-0.620). In contrast, %MA and leg length discrepancy were not significantly associated with the inter-ankle height difference in the multivariable models (Table 3).

**Table 3:** Results of univariate and multivariate regression analyses showing the relationship between the inter-ankle height difference and the other coronal spinopelvic and lower limb alignments in patients with Crowe type IV DDH (n = 87) CI, confidence interval; DDH, developmental dysplasia of the hip; β, regression coefficient ^a^Adjusted for age (years, continuous), sex (0: male, 1: female), and body mass index (kg/m², continuous)

Parameter	Crude β	95% CI	p-value	Adjusted β^a^	95% CI	p-value
%MA	0.097	-0.064 to 0.257	0.234	-0.036	-0.179 to 0.107	0.620
Leg length discrepancy	-0.079	-0.374 to 0.215	0.594	-0.094	-0.339 to 0.151	0.447
Pelvic inclination	1.473	0.824 to 2.123	<0.001	1.100	0.532 to 1.669	<0.001
Cobb angle	0.400	0.053 to 0.748	0.024	0.330	0.039 to 0.620	0.027

## Discussion

The present study investigated compensatory mechanisms of coronal spinopelvic and lower limb alignment in patients with unilateral Crowe type IV DDH. The key findings were that ankle-level compensation, represented by the inter-ankle height difference, was significantly associated with pelvic inclination, and that spinal alignment showed an association with ankle-level compensation in multivariable analysis, whereas knee alignment showed no robust association. 

In patients with unilateral Crowe type IV DDH, long-standing leg length discrepancy is accommodated through a series of compensatory mechanisms. More specifically, ankle-level compensation, potentially including equinus positioning, directly alters the functional length of the lower limb and may reduce the need for compensatory pelvic obliquity [3]. Because the pelvis serves as a central junction between the lower limbs and the spine, alterations at the ankle level are likely to be transmitted primarily to pelvic alignment [3]. The significant correlation observed between pelvic inclination and the Cobb angle in the present study and a previous study [6] further supports the concept that pelvic alignment mediates the relationship between distal compensation and spinal alignment. In contrast, knee alignment may be less sensitive to ankle-level compensation, as it is predominantly influenced by load distribution and frontal plane moments rather than by vertical adjustments in leg length [11-13]. These findings suggest that compensatory mechanisms for long-standing leg length discrepancy may preferentially involve the pelvis as the primary site of adjustment, with secondary adaptation of the spine.

The present results have important clinical implications. Ankle-level compensation is associated with pelvic obliquity and spinal scoliosis, suggesting that long-term external leg length correction, such as shoe lifts, may influence spinopelvic compensatory demands. In patients who already exhibit substantial ankle-level compensation, uniform application of shoe lifts may potentially alter established compensatory balance and may increase mechanical demand on the pelvis or spine. In contrast, in patients with limited ankle-level compensation, leg length correction may reduce the need for proximal compensatory mechanisms. Therefore, the biomechanical effects of shoe lifts are likely to depend on individual compensatory patterns, indicating that a uniform approach to leg length correction may not be appropriate for all patients with unilateral Crowe type IV DDH. Because of the cross-sectional design of this study, these clinical implications may be interpreted as hypothesis-generating rather than causal. Further longitudinal studies are needed to clarify these hypotheses.

There are some limitations in this study. First, because of the cross-sectional design, causal relationships and temporal sequences of compensatory mechanisms could not be determined. Second, we did not assess joint pain severity or range of motion in individual joints, which may influence compensatory patterns. Third, coronal alignment was evaluated using only standing anteroposterior radiographs, and sagittal and axial alignment were not assessed. In addition, compensatory mechanisms related to leg length discrepancy may also involve sagittal plane adaptations, such as knee flexion, which were not evaluated in the present study. Furthermore, the use of shoe lifts or other external leg length correction devices was not systematically assessed, and such interventions may have influenced compensatory alignment patterns, potentially masking ankle-level compensation. Fourth, dynamic alignment was not evaluated using two-dimensional gait analysis or three-dimensional motion analysis; therefore, static alignment assessed in the present study may differ from alignment during walking or other functional activities. Finally, this was a single-center study with a relatively limited sample size, although unilateral Crowe type IV DDH represents an uncommon condition in contemporary clinical practice [2]. While future longitudinal studies may further improve our understanding of compensatory mechanisms, the rarity of neglected DDH - often corresponding to Crowe type IV DDH [2] - in contemporary clinical practice limits the feasibility of such studies. Accordingly, the present study provides valuable cross-sectional evidence based on detailed radiographic evaluation in this unique patient population.

## Conclusions

This cross-sectional study demonstrated that ankle-level compensation, reflected by the inter-ankle height difference, was associated with coronal pelvic inclination and coronal spinal alignment in patients with unilateral Crowe type IV DDH, whereas no clear association was observed with coronal knee alignment. These findings suggest that compensatory alignment changes for long-standing leg length discrepancy may not be uniformly distributed across joints but are preferentially manifested at the pelvic level, with secondary involvement of the spine rather than the knee. Although causal relationships cannot be established because of the cross-sectional design, this study provides further insight into coronal compensatory alignment characteristics.
